# Food intake in an Australian Aboriginal rural community facing food and water security challenges: A cross‐sectional survey

**DOI:** 10.1111/1747-0080.12902

**Published:** 2024-09-25

**Authors:** Emalie Rosewarne, Trish Tonkin, Alinta Trindall, Joseph Alvin Santos, Dori Patay, Ruth McCausland, Wendy Spencer, Christine Corby, Julieann Coombes, Tamara Mackean, Greg Leslie, Niall Earle, Eileen Baldry, Janani Shanthosh, Ty Madden, Ann‐Marie Deane, Loretta Weatherall, Bruce Moore, Keziah Bennett‐Brook, Jacqui Webster

**Affiliations:** ^1^ The George Institute for Global Health University of New South Wales Sydney New South Wales Australia; ^2^ Dharriwaa Elders Group Walgett New South Wales Australia; ^3^ Yuwaya Ngarra‐li partnership University of New South Wales Sydney New South Wales Australia; ^4^ Department of Business Economics, Health and Social Care (DEASS) University of Applied Sciences and Arts of Southern Switzerland (SUPSI) Manno Switzerland; ^5^ Walgett Aboriginal Medical Service Limited Walgett New South Wales Australia; ^6^ College of Medicine and Public Health Flinders University Adelaide South Australia Australia; ^7^ Global Water Institute University of New South Wales Sydney New South Wales Australia; ^8^ Faculty of Law and Justice University of New South Wales Sydney New South Wales Australia

**Keywords:** Australian Aboriginal and Torres Strait islander peoples, dietary guidelines, food intake, nutrition requirements, rural and remote communities

## Abstract

**Aims:**

Researchers were invited by Aboriginal leaders to collaborate on this study which aimed to assess food intake in the Walgett Aboriginal community to inform long‐term community‐led efforts to improve food and water security and nutrition.

**Methods:**

Aboriginal adults living in or near Walgett, a remote community in north‐west NSW, Australia, completed an adapted Menzies Remote Short‐item Dietary Assessment Tool, which was administered verbally and face‐to‐face in early 2022. Aboriginal people were involved in the survey design, training and collection, and analysis of data. Descriptive statistics were tabulated, overall and by gender, age, and location. Differences by sex, age group (18‐44 years versus ≥45 years), and location (Walgett town or other) were determined using a chi‐square test.

**Results:**

A total of 242 participants completed the survey; 55% were female. Three‐quarters of participants reported meeting the recommendations for discretionary foods (73%); however, more than half (56%) exceeded the recommended maximum serves of sugar‐sweetened beverages. The proportion of participants meeting core food group guidelines was 72% for meat, 36% for fruit, 20% for bread and cereals, 6% for dairy, and 3% for vegetables. Overall, none of the participants met the recommended serves of all food groups outlined in the Australian Dietary Guidelines.

**Conclusion:**

Findings show that Walgett Aboriginal community members surveyed were consuming a healthier diet than national data reported for Aboriginal and Torres Strait Islander people in Australia. However, none of the participants were meeting all of the national dietary guidelines, placing them at increased risk of diet‐related chronic disease. Local Aboriginal community‐led efforts to improve food and water security should include specific strategies to improve nutrition.

## 1 INTRODUCTION

For millennia, Aboriginal and Torres Strait Islander Peoples thrived by consuming traditional diets, rich in local flora and fauna including native fruits and vegetables, lean land animals (e.g., kangaroo), and seafood, that varied based on seasons and geographic location.^1^, ^2^, ^3^, ^4^ European settlement in the late 1800s disrupted Aboriginal and Torres Strait Islander peoples' connection to Country, including traditional food and water systems.^1^, ^2^ This disconnection, and the introduction of Western foods, resulted in a rapid nutrition transition from traditional diets high in fibre, protein, poly‐unsaturated fats, and slowly digestible carbohydrates towards more Western‐style diets high in refined carbohydrates, added sugars, saturated fats, and salt, and low in fibre.^1^, ^4^, ^5^, ^6^, ^7^ This shift in food and nutrient intake resulted in an increase in diet‐related chronic diseases experienced by Aboriginal and Torres Strait people.^1^ A range of socioeconomic, environmental, and geographic factors including lower incomes and less access to healthy and affordable food contribute to the ongoing food and water security, nutrition, and health disparities between Aboriginal and Torres Strait Islander people and the non‐Indigenous population.^1^, ^4^, ^8^, ^9^ Almost 10% of the burden of disease for Aboriginal and Torres Strait Islander people stems from dietary risk factors.^1^

There is little high‐quality food intake data available for Aboriginal and Torres Strait Islander communities across Australia. There have been limitations in national data collection methods due to resource and geographical constraints, challenges in identifying culturally appropriate dietary assessment methods, and difficulties estimating food intake from the methods used.^1^, ^6^, ^10^ National surveys, such as the National Aboriginal and Torres Strait Islander Nutrition and Physical Activity Survey, provide some insight into Aboriginal and Torres Strait Islander peoples nutrition.^11^ In particular, Aboriginal and Torres Strait Islander peoples are generally not meeting the minimum recommended intake of the five core food groups (fruit, vegetables, grains, dairy and alternatives, and meat and alternatives) and are exceeding the recommended maximum intake of discretionary foods.^11^ Foods consumed can vary greatly between communities depending on environmental and geographic factors.^5^, ^12^ In many Aboriginal and Torres Strait Islander communities, fruit, and vegetable intakes are below the recommended minimum amounts, sugar, and sodium intakes exceed the recommended maximum amounts, and energy‐dense, nutrient‐poor foods are cheap, convenient, and easily accessed.^10^ However, more accurate food intake data are essential for planning, implementing, and evaluating community‐led initiatives in Aboriginal communities.^10^

Walgett is a remote community in NSW with a population of ~2200 people of whom almost half identify as Aboriginal according to the Australian Bureau of Statistics.^13^ Aboriginal people living in Walgett have a strong connection to Country, and local Aboriginal community‐controlled organisations advocate for a holistic concept of well‐being that embeds this connection in all their work. Walgett is located near agricultural land and at the junction of two rivers.^14^, ^15^ The Walgett community have faced several food and water security challenges in recent years including drought and floods, as well as the local supermarket burning down (the next closest supermarket is 80 km away), the river being dried up (no fish, not palatable for drinking), and high‐sodium bore water being the main source of town water.^3^, ^15^, ^16^, ^17^, ^18^, ^19^, ^20^ These food and water security challenges have impacted the community's ability to access healthy foods and consume a healthy, balanced diet. In view of recent challenges, local Aboriginal community‐controlled organisations have prioritised the need to establish resilient food and water systems to ensure community members can access affordable, nutritious food and safe drinking water.^15^, ^16^ Strengthening food and water security in Walgett will help to improve nutrition and reduce rates of chronic disease.^15^, ^16^ Since 2019, as part of a long‐term partnership between the Dharriwaa Elders Group and the University of New South Wales, an innovative community‐led initiative to improve food and water security encompassing Indigenous rights and knowledges has been developed and is now being implemented.^15^ The initiative includes installing reverse‐osmosis drinking water kiosks, creating a healthier supermarket, and directing more resources to the community garden.^15^

The aim of this study is to assess food intake in the Walgett Aboriginal population as part of a broader study to improve food and water security. The data will be used as a baseline measurement to inform and monitor the impact of the community‐led initiative over time.

Note this article mainly refers to Aboriginal people rather than Aboriginal and Torres Strait Islander people, Indigenous people, or First Nations, in keeping with common usage in the Walgett community. We recognise that Aboriginal and Torres Strait Islander peoples have unique cultures, histories, beliefs, and values. When referring to other literature, we utilise the terminology used in the source including referring to Aboriginal and Torres Strait Islander people or Indigenous people. The term ‘food intake’ is used rather than ‘dietary intake’ (where possible) to acknowledge the connectedness between Aboriginal people, Country and food systems. The authors recognise that food intake is just one component of Aboriginal health and well‐being, which is a holistic concept encompassing physical, social, emotional, cultural, and spiritual well‐being and connection to Country, and this study must be interpreted within this framing.

## 2 METHODS

Aboriginal people in a remote community in Australia completed a food intake survey in early 2022. Participants were compensated for their time. Aboriginal people were involved in the study design (grant writing/development of study protocols), training and data gathering (5/8 survey team members were Aboriginal), analysis of data and writing of the paper (writing workshops/contributing to drafts). This work was done in accordance with the STROBE guidelines for observational studies.

An intensive two‐day training and planning session took place immediately prior to the survey and the survey team reported back daily throughout the survey to share lessons and ensure the tools were being used consistently. Researchers were trained to use the questionnaire alongside the picture photobook (containing images of foods: for example, red meats—beef mince, kangaroo steak, canned processed red meat) and serving size prompts (e.g., hand with palm circled to indicate one serve of red meat) to assist with respondent estimation of serving size and consumption frequency.

Potential participants were Aboriginal people, aged 18 years and over, or their non‐Aboriginal family members, living in and near Walgett. Participants were recruited through convenience sampling, with an equal number of participants of each sex and age group aiming to be recruited (i.e., male/female, 18–44 years/45 years and older). We aimed to recruit ~250 people which is about one quarter of the Aboriginal population in Walgett. This sample size was adequate to achieve an 80% power to detect a 10% difference in the proportion of participants meeting the Australian Dietary Guidelines by food group, assuming the survey would be repeated with the same set of participants, with a presumed correlation of 0.4 between repeated measurements. The local supermarket granted permission for the research team to approach potential participants entering or leaving the supermarket and invite them to participate in the study. Individuals who agreed to participate completed consent forms.

The paper‐based survey (File S1) was administered face‐to‐face in Walgett town in April 2022. Researchers interviewed the participants outside the supermarket in Walgett Aboriginal Medical Service tents or across the road in the Dharriwaa Elders Group offices. Participants who completed the survey were provided with a $25 voucher that could be used in local shops including the supermarket, the butchers, or the hairdressers.

The food intake survey was adapted from the Menzies Remote Short‐Item Dietary Assessment Tool—a low‐burden, short‐form food frequency questionnaire that can be used to estimate usual food intake retrospectively, based on what participants eat either per day or per week, depending on the food, and compare intakes to the Australian Dietary Guidelines. The tool has been validated in Aboriginal mothers and children in remote communities in Australia.^21^, ^22^ It comprised 32 questions on types and frequency of foods and drinks consumed including core foods (fruit, vegetables, dairy, bread and cereals, and meat), discretionary foods and sugar‐sweetened beverages, and traditional foods (e.g., native flora and fauna). Adaptations were made based on community consultation, for example, modifying the example foods in the survey to include local and traditional foods (e.g., Local fruits, nuts, or other plants) and traditional fish or meat including turtle, crayfish, yabbies, emu, kangaroo, goanna. Culturally appropriate questions (*n* = 7) about socio‐demographic factors were developed collaboratively by the research team. Participants were given the option to not answer the questions they did not feel comfortable answering. The tools were pilot tested with six community members attending the Dharriwaa Elders Group before use to check acceptability and language usage—no changes were made. Data were recorded by the research team member using pen and paper.

Descriptive statistics (mean, SD, *n*, %) of participant characteristics were tabulated overall and by gender. The mean (SD) serves of each food group per day, were calculated overall and by gender, age group, and location. The proportion of participants meeting the Australian Dietary Guidelines by food group was calculated overall and by the age and gender classifications specified in the Guidelines. STATA IC version 16.0 for Windows (StataCorp LP, College Station, TX, USA) was used to conduct the analysis. Differences by sex, age group (18‐44 years versus ≥45 years), and location (Walgett town or other) were determined using a chi‐square test. Figures were generated in R version 1.2.1572.^23^

This study was approved by the Aboriginal Health and Medical Research Council of New South Wales (1781/20) and was ratified by the University of New South Wales Human Research Ethics Committee. This research was performed as per the Declaration of Helsinki. Verbal or written informed consent was obtained from all participants before the survey.

Aboriginal community members, including partner organisations and researchers, have been involved in the study design, gathering, and analysis of data while also ensuring privacy and ethics requirements are met. Management of data was consistent with Dharriwaa Elders Group and the University of New South Wales Yuwaya Ngarra‐li partnership principles and protocols.^24^

## 3 RESULTS

A total of 242 participants completed both surveys: 55% were female and 53% were aged 45 years or older (Table 1). Almost all participants identified as an Aboriginal person (97%) and the other six participants were living with, or caring for, an Aboriginal person so the team agreed that they should be allowed to participate. The majority of the participants lived in Walgett town (80%), had completed secondary level education (75%), lived in a household of two to five people (66%) who usually ate together (65%), and were the primary shopper and cook for the household (57%).

**TABLE 1:** Participant characteristics, overall and by gender.

Characteristics	Overall (*n* = 242)	Males (*n* = 109)	Females (*n* = 133)
Age, years (mean, SD)	45.5 (15.8)	45.8 (15.2)	45.2 (16.4)
Age group (*n*, %)			
18–44 years	113 (46.7)	50 (45.9)	63 (47.4)
45 years and up	129 (53.3)	59 (54.1)	70 (52.6)
Aboriginal origin (*n*, %)			
No	6 (2.5)	1 (0.9)	5 (3.9)
Yes	233 (97.5)	108 (99.1)	125 (96.2)
Location (*n*, %)			
Walgett Town	194 (80.2)	85 (78.0)	109 (82.0)
Gingie Reserve/Namoi Village/Out of town Walgett area	46 (19.0)	22 (20.2)	24 (18.1)
Unknown	2 (0.8)	2 (1.8)	0 (0.00)
Highest level of education (*n*, %)*			
Primary level	23 (9.6)	10 (9.3)	13 (9.9)
Secondary level	180 (75.3)	89 (82.4)	91 (69.5)
Tertiary level	35 (14.6)	9 (8.3)	26 (19.9)
Postgraduate or higher	1 (0.4)	0 (0.0)	1 (0.8)
How many people do you usually live with? (*n*, %)			
Live alone	23 (9.5)	14 (12.8)	9 (6.8)
Live with partner	23 (9.5)	11 (10.1)	12 (9.0)
Shared household (2–5 people)	160 (66.1)	72 (66.1)	88 (66.2)
Shared household (6–8 people)	35 (14.5)	12 (11.0)	23 (17.3)
Shared household (>8 people)	1 (0.4)	0 (0.0)	1 (0.8)
How many people do you usually eat with? (*n*, %)			
Eat alone	23 (9.1)	13 (11.9)	9 (6.8)
Eat with partner	20 (8.3)	10 (9.2)	10 (7.6)
Shared household (2–5 people)	157 (65.2)	72 (66.1)	85 (64.4)
Shared household (6–8 people)	38 (15.8)	13 (11.9)	25 (18.9)
Shared household (>8 people)	4 (1.7)	1 (0.9)	3 (2.3)
Which of the following best describes your role? (*n*, %)*			
Responsible for shopping and cooking most of the time	134 (56.8)	46 (42.6)	88 (68.8)
Responsible for shopping most of the time	6 (2.5)	3 (2.8)	3 (2.3)
Responsible for cooking most of the time	2 (0.9)	1 (0.9)	1 (0.8)
Share responsibility for shopping and cooking	73 (30.9)	42 (38.9)	31 (24.2)
Not responsible for shopping and cooking/eat with family most of the time	21 (8.9)	16 (14.8)	5 (3.9)

* Significant difference by sex at *p* < 0.05.

**FIGURE 1:**
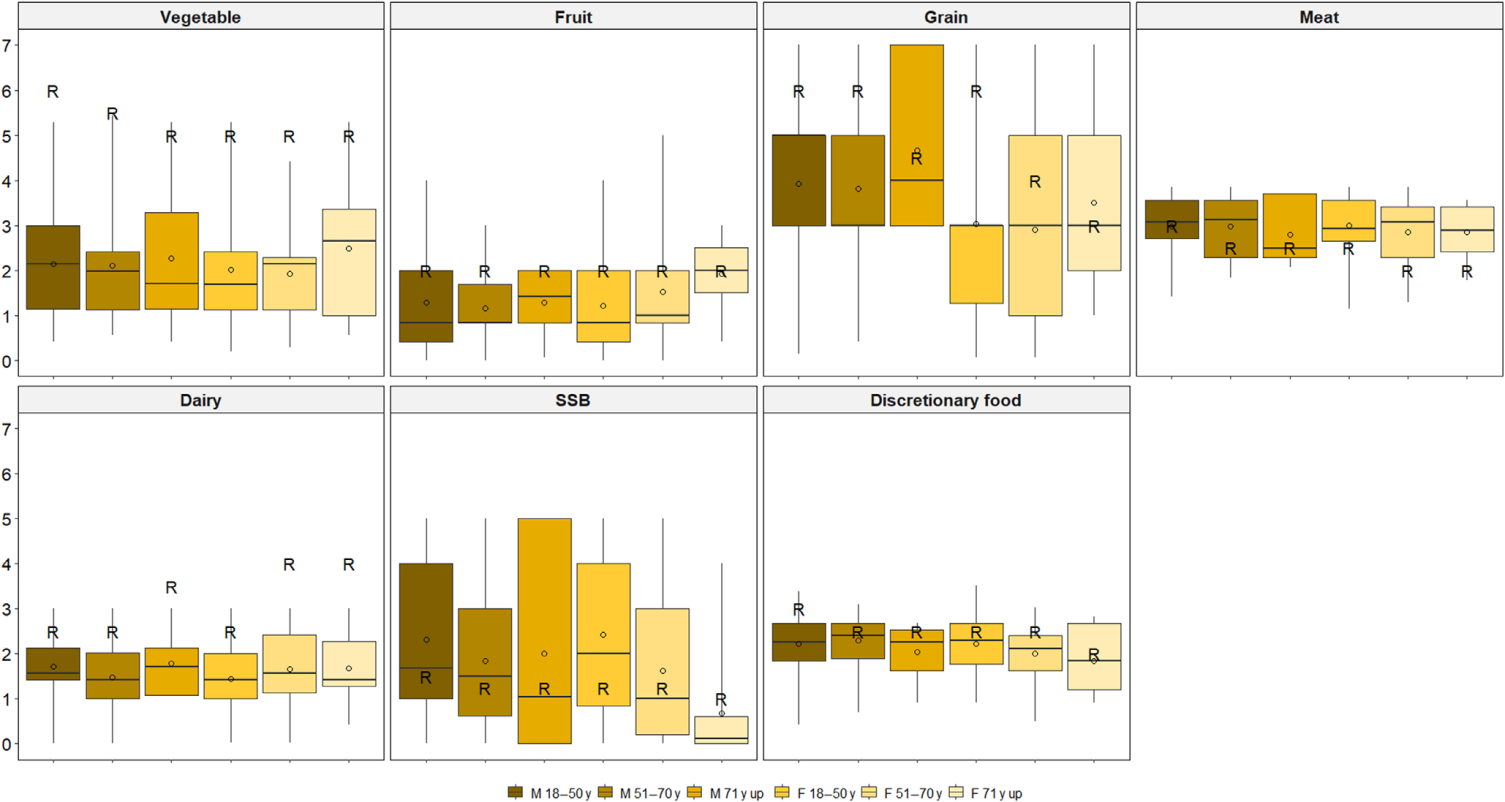
Distribution of serves per day for each food category calculated from the Menzies Remote Short‐item Dietary Assessment Tool, by Australian Dietary Guidelines classification group. The circle represents mean. R represents recommended serves.

Food intake assessment calculated using the Menzies Remote Short‐Item Dietary Assessment Tool found that, overall, none of the participants were meeting the recommended serves of food groups outlined in the Australian Dietary Guidelines. Positively, three‐quarters (73%) of the sample reported consuming less than the recommended maximum serves of discretionary foods; however, 56% exceeded the maximum recommended amount of sugar‐sweetened beverages (Table 2). The proportion of participants meeting core food group guidelines were: 72% for meat, 36% for fruit, 20% for bread and cereals, 6% for dairy, and 3% for vegetables. Figure 1 illustrates the distribution of estimated serves per day for each food category calculated from the Menzies Remote Short‐Item Dietary Assessment Tool, by the Australian Dietary Guidelines' classification group.

**TABLE 2:** Proportion of sample meeting the recommended number of serves of food groups calculated from the Menzies Remote Short‐Item Dietary Assessment Tool.

Food group^a^	Total (%)	Males						Females					
		18–50 years (*n* = 65)		51–70 years (*n* = 38)		71 years and up (*n* = 6)		18–50 years (*n* = 83)		51–70 years (*n* = 42)		71 years and up (*n* = 8)	
		*R*	*n* (%)	*R*	*n* (%)	*R*	*n* (%)	*R*	*n* (%)	*R*	*n* (%)	*R*	*n* (%)
Vegetables	2.5	≥6	0 (0.0)	≥5.5	0 (0.0)	≥5	1 (16.7)	≥5	4 (4.9)^b^	≥5	0 (0.0)	≥5	1 (12.5)
Fruit	36.3	≥2	25 (38.5)	≥2	9 (23.7)	≥2	3 (50.0)	≥2	25 (30.9)^b^	≥2	19 (45.2)	≥2	6 (75.0)
Bread and cereals	20.1	≥6	13 (20.0)	≥6	6 (15.8)	≥4.5	3 (50.0)	≥6	9 (11.1)^b^	≥4	11 (26.8)^b^	≥3	6 (75.0)
Meat	72.3	≥3	37 (57.8)^b^	≥2.5	26 (68.4)	≥2.5	3 (50.0)	≥2.5	65 (80.3)^b^	≥2	34 (82.9)^b^	≥2	7 (87.5)
Dairy^c^	5.8	≥2.5	8 (12.5)^b^	≥2.5	2 (5.3)	≥3.5	0 (0.0)	≥2.5	4 (4.9)^b^	≥4	0 (0.0)	≥4	0 (0.0)
Sugar‐sweetened drinks^d^	43.9	<1.5	23 (36.5)^b^	<1.25	18 (47.4)	<1.25	3 (50.0)	<1.25	29 (36.3)^b^	<1.25	25 (59.3)^b^	<1	6 (75.0)
Discretionary foods	73.4	<3	58 (92.1)^b^	<2.5	23 (62.2)^b^	<2.5	4 (66.7)	<2.5	48 (60.0)^b^	<2.5	34 (85.0)^b^	<2	4 (57.1)^b^

Abbreviation: R, recommended average daily number of serves.

^a^ According to the Australian Dietary Guidelines.

^b^ Number of serves could not be calculated for at least one participant.

^c^ Assumptions were made when calculating the recommended number of serves of dairy due to limitations in the questions asked (e.g., when participants answered yes to drinking plain milk every day, this was assumed to be one serve), likely resulting in an underestimation.

^d^ Recommended serves for sugar‐sweetened drinks and discretionary foods were based on the criteria for different age groups as outlined in McNaughton et al.^38^

The mean number of serves per day for each food group were approximately: 3 serves of bread and cereals, and meat, 2 serves of vegetables, sugar‐sweetened beverages, and discretionary foods, 1.5 serves of dairy, and 1 serve of fruit (Table 3; the median number of serves per day are reported in File S2). There were minimal differences in the mean number of serves consumed by sex, age, and location. Differences included male participants consuming more bread and cereals than females, younger participants consuming more sugar‐sweetened beverages and discretionary food than older participants, and participants outside of Walgett town consuming more meat than Walgett town residents (all *p* < 0.05).

**TABLE 3:** Mean (SD) serves per day of each food group estimated from the Menzies Remote Short‐Item Dietary Assessment Tool.

Food group	Overall	By sex		By age group		By location^a^	
		Males	Females	18–44 years	45 years and up	Walgett town	Other areas^b^
Vegetables (*n* = 237)	2.1 (1.2)	2.1 (1.3)	2.0 (1.2)	2.0 (1.2)	2.1 (1.3)	2.1 (1.3)	1.8 (0.9)
Fruit (*n* = 240)	1.3 (1.0)	1.2 (0.9)	1.4 (1.0)	1.2 (1.0)	1.4 (1.0)	1.2 (1.0)	1.6 (1.0)
Bread and cereals (*n* = 239)*	3.4 (2.1)	3.9 (2.1)	3.0 (2.1)	3.6 (2.2)	3.3 (2.1)	3.4 (2.2)	3.7 (1.9)
Meat (*n* = 238)***	3.0 (0.6)	3.0 (0.6)	2.9 (0.6)	3.0 (0.6)	2.9 (0.7)	2.9 (0.6)	3.2 (0.6)
Sugar‐sweetened drinks (*n* = 237)**	2.1 (1.8)	2.1 (1.8)	2.1 (1.8)	2.6 (1.8)	1.7 (1.7)	2.1 (1.8)	2.0 (1.9)
Discretionary foods (*n* = 233)**	2.2 (0.6)	2.2 (0.6)	2.1 (0.6)	2.4 (0.6)	2.0 (0.6)	2.2 (0.6)	2.3 (0.6)
Dairy (*n* = 240)	1.6 (0.8)	1.6 (0.8)	1.5 (0.8)	1.6 (0.8)	1.6 (0.8)	1.6 (0.8)	1.6 (0.6)

^a^ Analysis by location excludes two respondents with unknown location.

^b^ Other areas include Gingie Reserve, Namoi Village, and out of town Walgett area.

* Significant difference by sex at *p* < 0.05.

** Significant difference by age group at p < 0.05.

*** Significant difference by location at *p* < 0.0.

Assessment of core and discretionary food intake showed around two‐thirds (64%) of participants reported consuming vegetables every day or nearly every day (File S3). Starchy, green‐coloured and orange/yellow/red‐coloured vegetables were consumed by more than 90% of participants. Less than half (44%) consumed fruit every day or nearly every day; the majority (56%) did not consume any fruit daily. Pome (e.g., apples), citrus, and tropical fruit were consumed by more than 80% of participants. For dairy foods, most participants (73%) consumed plain milk nearly every day and cheese or yoghurt at least a few times a week (74%).

Most participants (78%) reported consuming bread and cereals every day or nearly every day; nearly half (46%) estimated they consumed two to three serves per day. More participants (73%) reported usually consuming white bread than wholegrain varieties (Table S2). The majority of participants reported consuming more than one serve of red meat (69%), processed meat (67%), and white meat (58%) per day. Some performed health‐promoting behaviours such as trimming the fat off red meat (51%) and taking the skin off chicken (32%).

Consumption of other protein sources was measured weekly: around half of the participants ate at least one serve of legumes (e.g., peas and beans; 47%) and fish (48%) per week, 81% ate at least 2 eggs per week, and 16% ate unsalted nuts at least once per week (Table S2). Around one‐fifth of survey participants ate traditional native flora (16%; e.g., fruit, nuts, other plants) and land animals (23%; e.g., fish, kangaroo, goanna) at least once a week; around half of the sample never ate traditional native flora and land animals (56% and 47%, respectively).

More than half of the group (55%) consumed sugar‐sweetened beverages every day or nearly every day with one fifth of the sample (20%) consuming more than 5 serves on a given day (Table S2). Discretionary food intake was low: around one‐quarter of participants reported consuming salty sauces or seasonings (26%), sweet snacks (25%), baked goods (21%), and savoury snacks (20%) three times a week or more. 10% reported consuming ready meals (12%) and take‐away (9%) three times a week or more.

There were several differences in reported food intake by sex, age and location (Table S2). Male participants were more likely to eat offal and beans, peas, or lentils; reported consuming greater quantities of bread and cereals; and were more likely to consume at least traditional meats and ready meals at least once per week, compared with females. Younger participants were less likely to eat orange/yellow/red vegetables and offal and more likely to eat sweetened yoghurt and white bread. People in Walgett town were more likely to eat berries and sweetened yoghurt, and trim the skin off chicken, than those living outside of Walgett town. There were differences by location in the frequency of consuming processed meat, sweet and savoury snacks, ready meals, fruit, vegetables, and legumes.

## 4 DISCUSSION

This study provides an overview of food intake in an Aboriginal community facing food and water insecurity challenges in remote Australia. Utilising a culturally appropriate tool that was validated in other Aboriginal communities, and minimally adapted here, enabled an estimation of individual‐level food intake, overcoming previously reported methodological barriers to understanding food intake in remote Aboriginal communities. The results must be contextualised within the larger narrative of Aboriginal health and well‐being, considering the persisting intergenerational impacts of historical events (e.g., European settlement, displacement, food rationing, shame around maintaining cultural practices and consuming traditional foods), more recent climate and health events (e.g., droughts, floods, and bushfires, and COVID‐19), and broader structural determinants (e.g., government policies, socio‐economic factors, geographical and environmental factors, and food and water insecurity) that have resulted in disconnection between Aboriginal people and Country and continue to impact food availability and choices.

Our findings suggest that the Walgett Aboriginal community are consuming a healthier diet than national data reported for Aboriginal and Torres Strait Islander people in Australia.^11^ Survey participants were, on average, consuming a greater number of serves of fruit (1.3 vs. 1.0), dairy (1.6 vs. 1.2), and meat (3.0 vs. 1.2), and much less discretionary food than the national averages for Aboriginal and Torres Strait Islander adults in Australia (4.3 serves of discretionary food and sugar‐sweetened beverages, compared with the national average of 6.1 serves).^11^ Meanwhile, bread and cereals intakes were slightly lower than the reported national average (3.4 vs. 4.1), and vegetable intake was the same (1.3).^11^ Additionally, compared with other remote Indigenous communities, Walgett area residents are consuming more vegetables, fruit, dairy, and meat.^11^ Furthermore, there was good variety in the fruit and vegetables consumed, with more than 80% of participants regularly consuming more than three variety‐types of both fruit and vegetables. While there are differences in the dietary assessment methodology between the current study and national surveys, both provide an estimate of usual intake and are therefore likely comparable.^11^, ^22^ Whilst further research is required to substantiate this, access to a relatively well‐stocked supermarket compared with more remote communities could be driving this difference in diet. Despite high levels of food and water insecurity, including fruit and vegetable affordability concerns, availability issues and an inability to grow fruit and vegetables due to water insecurity and other environmental impacts,^25^ Walgett Aboriginal community members surveyed are consuming a healthier diet than other Aboriginal and Torres Strait Islander people in Australia.^11^

Although these findings are positive, the majority of the community's food intakes were still not meeting the recommended amounts of four out of five core food groups outlined in national dietary guidelines^8^ including fruit, vegetables, breads and cereals, and dairy, placing the community at risk of diet‐related diseases. This is consistent with surveys conducted in other Aboriginal communities and national data.^10^, ^11^ There has not been a national nutrition strategy for Aboriginal and Torres Strait Islander peoples since the previous strategy lapsed in 2010.^1^ What's more, this Government‐led approach was largely unsuccessful, as community‐controlled organisations were not involved in the design, and implementation was not wide‐spread or well resourced.^26^ On the other hand, initiatives where communities are involved in, and have control of, program development, implementation, and evaluation are more likely to be successful^27^ as strategies to improve food intake can then be tailored to community‐identified needs.^28^ The results of this survey further confirm the need for community‐led initiatives, such as that being implemented in Walgett, to improve food intake.

In line with findings from other communities, consumption of sugar‐sweetened beverages was high and this is associated with negative health impacts.^29^ The high frequency and amount of sugar‐sweetened beverages consumed (>50% reporting ≥2 serves every day/nearly every day) is likely due to several factors being experienced by Aboriginal people in Walgett and other remote communities around Australia including degradation of river systems, and community concerns around the safety and palatability of the town water and the relatively cheaper cost of sugar‐sweetened beverages compared with bottled water.^15^, ^30^, ^31^, ^32^, ^33^ With around one‐fifth of survey participants reporting consuming more than five serves on a given day, it is likely a proportion of community members are accessing sugary drinks as an alternate hydration source to town water, increasing their risk of diet‐related diseases, including obesity. The community‐led initiative aims to increase the community's access to safe, good‐quality, and palatable drinking water through the installation of reverse‐osmosis drinking water kiosks. In addition, the Walgett Aboriginal Medical Service and Dharriwaa Elders Group are advocating for immediate government action to improve town water quality, which will reduce community reliance on sugary drinks as a hydration source.

Around one‐fifth of survey participants reported eating traditional native plants and land animals at least once a week. This finding highlights that Aboriginal people living in and near Walgett are able to access traditional land foods. Additionally, it may be that up to 52% of participants consume wild‐caught fish and yabbies weekly; however, there were limitations in the design of our survey question that meant we were unable to distinguish between wild‐caught and purchased seafood. Overall, the proportion of the Walgett Aboriginal community consuming traditional foods is likely much higher than nationally reported estimates for Aboriginal and Torres Strait Islander peoples living in remote areas, which are estimated to be <8% for wild‐caught fish and meat, 4% reptiles, amphibia, and insects, and 0.3% wild‐harvested native flora.^11^ However, the estimated weekly consumption of traditional foods from our study was lower than that of remote Aboriginal communities in the Northern Territory, where 70% of the community reported consuming traditional plants and animals weekly.^34^ The fact that around half of participants from the Walgett study reported never eating traditional native plants and land animals, illustrates the greater impact of the degradation of Country in that area. The Walgett Aboriginal community have experienced the loss of fishing for traditional foods due to river degradation, mismanagement, and drought, and the loss of plant food due to land clearing for agriculture, drought, limited access to land, and an inability to maintain gardens because of high‐sodium bore water.^15^ These losses not only impact food intake, nutrition and physical health, but also the social, cultural and emotional health and well‐being of the Aboriginal community.^31^ The reconnection between Aboriginal people, Country, and culture, including privileging Indigenous knowledges around healthy and sustainable food and water systems, is key to improved food intake, nutrition and health in Aboriginal communities.

This research has estimated the food group intakes in the Walgett Aboriginal community in remote New South Wales, Australia. The findings have significant implications for policy and practice and reinforce the need for high‐quality, culturally appropriate food intake assessments in rural and remote Aboriginal and Torres Strait Islander communities across Australia, despite the greater resources required and challenges faced in collecting data.^1^, ^6^, ^10^ This will enable community‐controlled organisations to lead tailored programs to improve community food intake based on local knowledge and data, such as further work to support community gardening, enabling the consumption of traditional plants and animals and healthy food retail strategies. These community‐led initiatives are more likely to be successful,^27^, ^28^ than current government‐led approaches that are not being widely implemented or well‐resourced.^26^ Instead, as proposed in the Federal Government consultation into food security in Australia, the government should focus on structural determinants, such as improving town water quality to reduce community reliance on sugary drinks as a hydration source, and fiscal measures and income supplements to lower the relative price of fresh, healthy foods and increase fruit and vegetable consumption.^33^, ^35^, ^36^, ^37^

An important strength of this study was that it was co‐designed with Walgett Aboriginal community‐controlled organisations and administered by a research team that included local Aboriginal community members. This contributed to a high participation rate (around one‐quarter of the Aboriginal population in Walgett) likely meaning that our results are representative of the broader community. However, we acknowledge a potential bias in that participants were rewarded with a food voucher, meaning people were more likely to participate if they were food insecure. The adapted dietary assessment tool was culturally appropriate and enabled the first estimation of individual‐level food intake in Walgett. Due to the adaptations a limitation was that we could not calculate an overall dietary assessment score; however, we were able to estimate food group intakes. There is good confidence in the estimates for all core and discretionary food groups except dairy (6/7) as the modifications to the questions led to several assumptions which likely resulted in an underestimation of actual intake. Additionally, this self‐reported data may be subject to participant bias (e.g., social desirability bias), the administration of the survey by the trained, local Aboriginal research team, likely minimised the magnitude of misreporting, and the dietary assessment tool has been previously validated for application in Aboriginal communities.

This study found that the Walgett Aboriginal community are consuming healthier diets than many other remote Aboriginal communities in Australia; however, national dietary guidelines are far from being met. To improve nutrition and health, this study confirms that local community‐led initiatives to improve food and water security should include specific strategies to support healthy food intakes, such as healthy food retail strategies, further work to support community gardening, and enabling the consumption of traditional plants and animals. Further research looking at the relationship between food and water security and diets (including soft drink consumption) needs to be undertaken. The planned government National Strategy for Food Security in Remote Aboriginal communities could be a good mechanism for addressing some of these issues but needs to build upon local community‐led programs to enact real change.

## AUTHOR CONTRIBUTIONS

*Conceptualization and methodology*: ER, JW, GL, RM, KBB, WS, CC, TM, and JS. Formal analysis: JAS, ER, and JW. Investigation: AT, TT, BM, AMD, TM, JW, LW, and NE. *Writing—original draft preparation*: ER, JW, and DP. *Writing—review and editing*: All authors. *Visualisation*: ER and JAS. *Project administration*: ER, TT, WS, JW, and AMD. *Funding acquisition*: JW, GL, RM, KBB, WS, CC, EB, JC, TM, and JS. All authors have read and agree the current version of the article which has not been published elsewhere.

This work was done under the Yuwaya Ngarra‐li ‘vision’ partnership between UNSW and the Dharriwaa Elders Group, in collaboration with the Walgett Aboriginal Medical Service (WAMS) and The George Institute for Global Health. Walgett Aboriginal Medical Service Ltd is an Aboriginal Community Controlled Health Organisation established in 1986 (walgettams.com.au) and Dharriwaa Elders Group (dharriwaaeldersgroup.org.au) is an Aboriginal Community Controlled Organisation established in 2000 by WAMS that became independent in 2005 and later established the Yuwaya Ngarra‐li partnership with UNSW from 2016. The authors would like to thank the community survey participants for their time and input to this report. The authors pay their respects to the custodians and Elders of the Gamilaraay, Ngayiimbaa, Wayilwan, and Yuwaalaraay nations in the Walgett region where the Dharriwaa Elders Group and Walgett Aboriginal Medical Service are based, and to the Gadigal and Bedegal People of the Eora Nation, who are the Traditional Owners of the lands where The George Institute for Global Health and UNSW campuses are situated.

## FUNDING INFORMATION

This research was funded by a National Health and Medical Research Council of Australia IDEAS grant (no. 2003862). Jacqui Webster is supported by a National Health and Medical Research Council of Australia Investigator Grant Level 2 (no. 2018015).

## CONFLICT OF INTEREST STATEMENT

The authors declare that they have no conflict of interests.

## Supporting information


**Supplementary Material**. Survey Tool.

**Table S1.** Median (IQR) serves per day of each food group estimated from the Menzies Remote Short‐Item Dietary Assessment Tool (MRSDAT).

**Table S2.** Frequencies and proportions of responses to each question using the adapted Menzies Remote Short‐Item Dietary Assessment Tool (MRSDAT).

## Data Availability

The data that support the findings of this study belong to the community but can be made available via the corresponding author upon reasonable request.
