# Supplementary material for: Food intake in an Australian Aboriginal rural community facing food and water security challenges: A cross‐sectional survey
Source: Nutr Diet. 2024 Sep 25;82(1):86–96. doi: 10.1111/1747-0080.12902 (PMC11795224; doi:10.1111/1747-0080.12902)
Supplement: Supplementary file 1 — Supplementary Material. Survey Tool. [file NDI-82-86-s003.docx]

**Adapted Menzies Remote Short-item Dietary Assessment Tool (MRSDAT)**

Introduction

The following questions ask about foods and drinks you consume. This survey is all about **some of the foods** that usually eat in a day and how often you eat these foods. There can be questions about certain food that you don’t eat and you can respond as **‘NONE’** to these questions. It is important to remember that each of us eats differently and so **there are no right or wrong answers** and we won’t be sharing your answers with anyone. There are example pictures of food types and amounts to help you to answer the questions. Please remember that the pictures are only examples and there can be other food types that you might eat.

Just a reminder that all information will be treated in confidence and anonymised in any reports.

Adapted Menzies Remote Short-item Dietary Assessment Tool

## 1.* Which of these vegetables do you usually eat?

Starchy vegetables (including potatoes, sweet potato, yam, Sweet corn, pumpkin etc.)

Green coloured vegetables

Orange/yellow/red coloured vegetables

Cooked, dried or canned beans, peas or lentils

Other coloured vegetables, please specify ………………………………………………….

I don’t usually eat vegetables (please go to Q4.)

## 2. How often do you eat vegetables?

*(Not including beans and lentils)*

Every day or nearly every day

A few times a week

Once a week

Once a fortnight

Once a month

## 3. On days you eat vegetables, how much do you usually eat in a day?

Refer to pictures with portion size

1 serve or less

2 serves

3 serves

4 serves

5 serves or more

## 4.* Which of these fruits do you usually eat?

Pome fruit e.g. apples and pears

Citrus fruit e.g. oranges, mandarins

Stone fruit e.g. apricots, peaches, cherries, plums

Tropical fruit e.g. bananas, paw paw, mangoes, pineapple

Berries

Other fruit, please specify

I don’t usually eat fruit (please go to Q7.)

## 5. How often do you eat fruit?

*(Includes freshly squeezed homemade juice)*

Every day or nearly every day

A few times a week

Once a week

Once a fortnight

Once a month

## 6. When you eat fruit, how much do you usually eat?

*Refer to portion size pictures*

1 serve or less

2 serves

3 serves

4 serves

5 serves or more

## 7. How often do you usually drink sugar sweetened drinks like soft drink, fruit drinks, added sugar fruit juice, cordials or sports drinks?

Every day or nearly every day

A few times a week

Once a week

Once a fortnight

Once a month

I don’t usually drink sugar sweetened drinks (go to Q 8)

## (7.b) How much do you usually drink?

Refer to serving sizes in pictures

None

0.5 serves or less

0.5-1 serve

1-1.5 serves

1.5-2 serves

2-2.5 serves

2.5-3 serves

3-5 serves

5+ serves

## 8. How often do you usually drink flavoured milk?

Every day or nearly every day

A few times a week

Once a week

Once a fortnight

Once a month

I don’t usually drink flavoured milk

## 9. How often do you usually have cheese or yoghurt?

Every day or nearly every day

A few times a week

Once a week

Once a fortnight

Once a month

I don’t eat usually cheese or yoghurt (go to Q10.)

## (9.b) If you have yoghurt, is it usually sweetened or unsweetened?

Sweetened/flavoured (vanilla-flavoured)

Unsweetened/plain

## 10. Do you drink plain milk nearly every day? (includes milk added with cereal)

Yes

No

Just in tea/coffee

## 11. How many serves of red meat (steak, beef, pork kangaroo, lamb, sausages) do you usually eat per day?

Less than 1 serve

1 serve or more

I don’t eat red meat (go to Q 13.)

## 12. When you eat red meat, do you usually cut the fat off?

Yes

No

## 13.* How many serves of processed meat (Devon, salami, sausage, ham, corned beef/pickled pork, rissole, smoked meat) do you usually eat per day?

Less than 1 serve

1 serve or more

I don’t eat meat

## 14. How many serves of white meats (chicken, turkey) do you usually eat per day?

Less than 1 serve

1 serve or more

I don’t eat white meat (go to Q16.)

## 15. When you eat chicken do you usually take the skin off?

Yes

No

## 16. Do you usually eat offal meat (such as kidney, liver, heart, brain, curly guts, moobal, tripe etc.)?

Yes

No

## 17. How much butter, margarine or cream do you usually eat per day?

Less than 1 serve

1 serve or more

## 18. How much baked beans, three beans mix, cooked lentils, split peas or dried beans do you usually eat per week? (1 serve is ½ cup cooked)

None

Less than 1 serve

1–2 serves

3 or more serves

## 19. How many eggs do you usually eat per week?

None

Less than 2 eggs

2–4 eggs

5or more eggs

## 20. How much fish including wild caught fish, fresh and frozen seafood including turtle, crayfish, yabbies, shrimp etc. do you usually eat per week? (excluding fish fingers or fish patties) (one serve is about 100g cooked or 115g raw)

None

Less than 1 serve

1–2 serves

3 or more serves

## 21. How many times per week do you usually eat traditional food (native fruits, berries, nuts, other wild plants, meats like fish, kangaroo, emu, goanna etc.)?

None

Less than 1 time

1–2 times

3 or more times

## 22. How many times per week do you usually eat unsalted nuts?

None

Less than 1 time

1–2 times

3 or more times

## 23. How many times per week do you usually eat pastries such as cookies/biscuits or cake?

None

Less than 1 time

1–2 times

3 or more times

## 24.* How many times per week do you usually eat savoury snacks (e.g. chips, pretzels)?

None

Less than 1 time

1–2 times

3 or more times

## 25.* How many times per week do you usually eat sweet snacks (e.g. lollies, chocolates or ice creams)?

None

Less than 1 time

1–2 times

3 or more times

## 26.* How many times per week do you usually eat store-bought ready meals (e.g. supermarket pizza, instant noodles)?

None

Less than 1 time

1–2 times

3 or more times

## 27. How many times per week do you usually eat meals or snacks from take-away such as pizza, burgers, fries, pies, fish and chips etc.?

None

Less than 1 time

1–2 times

3 or more times

## 28.* How many times per week do you usually use salty seasonings/sauces for vegetables, pasta, rice, or other dishes?

None

1 time

2 or more times

## 29. How often do you eat bread and cereals (breakfast cereals, bread, scones, jonny cakes, damper, rice, pasta, other grains)?

Every day or nearly every day

A few times a week

Once a week

Once a fortnight

Once a month

I don’t eat bread and cereals (end survey)

## 30. When you eat bread and cereals, how much do they usually eat per day?

1 serve or less

2–3 serves

4–5 serves

6–7 serves

7 serves or more

## 32. What type of bread do you usually eat?

None

White

Multigrain/rye/wholemeal

Thanks for your time!
