# Supplementary material for: Food intake in an Australian Aboriginal rural community facing food and water security challenges: A cross‐sectional survey
Source: Nutr Diet. 2024 Sep 25;82(1):86–96. doi: 10.1111/1747-0080.12902 (PMC11795224; doi:10.1111/1747-0080.12902)
Supplement: Supplementary file 2 — Table S1. Median (IQR) serves per day of each food group estimated from the Menzies Remote Short‐Item Dietary Assessment Tool (MRSDAT). [file NDI-82-86-s002.docx]

**Supplementary Table 1** Median (IQR) serves per day of each food group estimated from the Menzies Remote Short-Item Dietary Assessment Tool (MRSDAT)

| **FOOD GROUP** | **OVERALL** | **BY SEX** | | **BY AGE GROUP** | | **BY LOCATION ^a^** | |
| --- | --- | --- | --- | --- | --- | --- | --- |
|  |  | **Males** | **Females** | **18 to 44 years** | **45 years and up** | **Walgett town** | **Other areas ^b^** |
| **Vegetables (*n* = 237)** | 2.00 (1.12 to 2.42) | 2.00 (1.12 to 3.00) | 2.00 (1.12 to 2.42) | 2.00 (1.12 to 2.42) | 2.07 (1.12 to 2.71) | 2.10 (1.14 to 3.00) | 1.40 (1.12 to 2.28) |
| **Fruit (*n* = 240)** | 1.00 (0.42 to 2.00) | 0.84 (0.42 to 2.00) | 1.00 (0.42 to 2.00) | 0.84 (0.42 to 2.00) | 1.00 (0.84 to 2.00) | 0.84 (0.42 to 2.00) | 1.63 (0.84 to 2.00) |
| **Bread and cereals (*n* = 239)** ^*^ | 3.00 (1.26 to 5.00) | 3.00 (3.00 to 5.00) | 3.00 (1.26 to 5.00) | 3.00 (1.26 to 5.00) | 3.00 (1.26 to 5.00) | 3.00 (1.26 to 5.00) | 3.00 (3.00 to 5.00) |
| **Meat (*n* = 238)** ^***^ | 3.06 (2.50 to 3.56) | 3.06 (2.56 to 3.56) | 3.06 (2.42 to 3.56) | 3.06 (2.64 to 3.56) | 3.06 (2.42 to 3.56) | 2.92 (2.42 to 3.56) | 3.38 (2.92 to 3.56) |
| **Sugar-sweetened drinks (*n* = 237)** ^**^ | 1.50 (0.63 to 4.00) | 1.50 (0.63 to 4.00) | 1.59 (0.42 to 4.00) | 2.00 (1.00 to 4.00) | 1.05 (0.21 to 3.00) | 1.68 (0.63 to 4.00) | 1.50 (0.56 to 4.00) |
| **Discretionary foods (*n* = 233)** ^**^ | 2.26 (1.84 to 2.68) | 2.26 (1.90 to 2.68) | 2.26 (1.70 to 2.54) | 2.46 (1.98 to 2.75) | 2.12 (1.62 to 2.40) | 2.26 (1.76 to 2.68) | 2.33 (1.84 to 2.68) |
| **Dairy (*n* = 240)** | 1.49 (1.11 to 2.07) | 1.56 (1.14 to 2.14) | 1.45 (1.03 to 2.03) | 1.56 (1.14 to 2.07) | 1.45 (1.07 to 2.03) | 1.56 (1.03 to 2.14) | 1.43 (1.14 to 2.00) |

^a^ Analysis by location excludes two respondents with unknown location

^b^ Other areas include Gingie Reserve, Namoi Village, and out of town Walgett area

^*^ Significant difference by sex at p<0.05.

^**^ Significant difference by age group at p<0.05.

^***^ Significant difference by location at p<0.05.
