# Supplementary material for: Food intake in an Australian Aboriginal rural community facing food and water security challenges: A cross‐sectional survey
Source: Nutr Diet. 2024 Sep 25;82(1):86–96. doi: 10.1111/1747-0080.12902 (PMC11795224; doi:10.1111/1747-0080.12902)
Supplement: Supplementary file 3 — Table S2. Frequencies and proportions of responses to each question using the adapted Menzies Remote Short‐Item Dietary Assessment Tool (MRSDAT). [file NDI-82-86-s001.docx]

**Supplementary Table 2** Frequencies and proportions of responses to each question using the adapted Menzies Remote Short-Item Dietary Assessment Tool (MRSDAT)

| **QUESTIONS** | **OVERALL**  (*n*=242) | **BY SEX** | | **BY AGE GROUP** | | **BY LOCATION ^a^** | |
| --- | --- | --- | --- | --- | --- | --- | --- |
|  |  | **Males**  (*n*=109) | **Females**  (*n*=133) | **18 to 44 years**  (*n*=113) | **45 years and up**  (*n*=129) | **Walgett town**  (*n*=194) | **Other areas ^b^**  (*n*=46) |
| **Which of these vegetables do you usually eat? (select as many as relevant)** | | | | | | | |
| Starchy vegetables (e.g. potato, sweet potato, yam, sweet corn) | 236 (97.52) | 107 (98.17) | 129 (96.99) | 110 (97.35) | 126 (97.67) | 188 (96.91) | 46 (100.00) |
| Green coloured vegetables | 233 (96.28) | 104 (95.41) | 129 (96.99) | 108 (95.58) | 125 (96.90) | 186 (95.88) | 46 (100.00) |
| Orange/yellow/red coloured vegetables ^**^ | 227 (93.80) | 100 (91.74) | 127 (95.49) | 102 (90.27) | 125 (96.90) | 181 (93.30) | 45 (97.83) |
| Cooked, dried or canned beans, peas or lentils ^*^ | 177 (73.14) | 89 (81.65) | 88 (66.17) | 86 (76.11) | 91 (70.54) | 143 (73.71) | 33 (71.74) |
| Other coloured vegetables ^***^ | 145 (59.92) | 63 (57.80) | 82 (61.65) | 65 (57.52) | 80 (62.02) | 110 (56.70) | 35 (76.09) |
| I don’t usually eat vegetables | 0 (0.00) | 0 (0.00) | 0 (0.00) | 0 (0.00) | 0 (0.00) | 0 (0.00) | 0 (0.00) |
| **How often do you eat vegetables (not including beans and lentils)?** | | | | | | | |
| Every day or nearly every day  A few times a week  Once a week  Once a fortnight  Once a month  I don’t usually eat vegetables | 153 (63.75)  80 (33.33)  5 (2.08)  1 (0.42)  1 (0.42)  0 (0.00) | 65 (60.75)  39 (36.45)  2 (1.87)  1 (0.93)  0 (0.00)  0 (0.00) | 88 (66.17)  41 (30.83)  3 (2.26)  0 (0.00)  1 (0.75)  0 (0.00) | 62 (55.86)  47 (42.34)  2 (1.80)  0 (0.00)  0 (0.00)  0 (0.00) | 91 (70.54)  33 (25.58)  3 (2.33)  1 (0.78)  1 (0.78)  0 (0.00) | 127 (65.80)  60 (31.09)  4 (2.07)  1 (0.52)  1 (0.52)  0 (0.00) | 25 (55.56)  19 (42.22)  1 (2.22)  0 (0.00)  0 (0.00)  0 (0.00) |
| **On days you eat vegetables, how much do you usually eat in a day? ^3^** | | | | | | | |
| 1 serve or less  2 serves  3 serves  4 serves  5 serves or more | 52 (21.67)  99 (41.25)  47 (19.58)  26 (10.83)  16 (6.67) | 21 (19.44)  42 (38.89)  20 (18.52)  15 (13.89)  10 (9.26) | 31 (23.48)  57 (43.18)  27 (20.45)  11 (8.33)  6 (4.55) | 17 (15.32)  50 (45.05)  25 (22.52)  13 (11.71)  6 (5.41) | 35 (27.13)  49 (37.98)  22 (17.05)  13 (10.08)  10 (7.75) | 44 (22.92)  69 (35.94)  40 (20.83)  24 (12.50)  15 (7.81) | 7 (15.22)  30 (65.22)  7 (15.22)  2 (4.35)  0 (0.00) |
| **Which of these fruits do you usually eat? (select as many as relevant)** | | | | | | | |
| Pome fruit (e.g. apples, pears) | 200 (82.64) | 92 (84.40) | 108 (81.20) | 97 (85.84) | 103 (79.84) | 162 (83.51) | 36 (78.26) |
| Citrus fruit (e.g oranges, mandarins) | 214 (88.43) | 96 (88.07) | 118 (88.72) | 100 (88.50) | 114 (88.37) | 173 (89.18) | 40 (86.96) |
| Stone fruit (e.g. apricots, peaches, cherries, plums) | 167 (69.01) | 75 (68.81) | 92 (69.17) | 72 (63.72) | 95 (73.64) | 139 (71.65) | 27 (58.70) |
| Tropical fruit (e.g. bananas, paw paw, mangoes, pineapples) | 213 (88.02) | 95 (87.16) | 118 (88.72) | 97 (85.84) | 116 (89.92) | 171 (88.14) | 41 (89.13) |
| Berries ^***^ | 140 (57.85) | 63 (57.80) | 77 (57.89) | 68 (60.18) | 72 (55.81) | 124 (63.92) | 15 (32.61) |
| Other fruits | 64 (26.45) | 27 (24.77) | 37 (27.82) | 27 (23.89) | 37 (28.68) | 56 (28.87) | 8 (17.39) |
| I don’t usually eat fruit | 5 (2.07) | 2 (1.83) | 3 (2.26) | 1 (0.88) | 4 (3.10) | 5 (2.58) | 0 (0.00) |
| **How often do you eat fruit (including freshly squeezed homemade juice)? ^2,3^** | | | | | | | |
| Every day or nearly every day  A few times a week  Once a week  Once a fortnight  Once a month  I don’t usually eat fruit | 105 (43.75)  104 (43.33)  20 (8.33)  4 (1.67)  2 (0.83)  5 (2.08) | 40 (36.70)  53 (48.62)  11 (10.09)  3 (2.75)  0 (0.00)  2 (1.83) | 65 (49.62)  51 (38.93)  9 (6.87)  1 (0.76)  2 (1.53)  3 (2.29) | 42 (37.50)  50 (44.64)  15 (13.39)  2 (1.79)  2 (1.79)  1 (0.89) | 63 (49.22)  54 (42.19)  5 (3.91)  2 (1.56)  0 (0.00)  4 (3.13) | 78 (40.63)  86 (44.79)  20 (10.42)  2 (1.04)  1 (0.52)  5 (2.60) | 26 (56.52)  17 (36.96)  0 (0.00)  2 (4.35)  1 (2.17)  0 (0.00) |
| **When you eat fruit, how much do you usually eat?** | | | | | | | |
| 1 serve or less  2 serves  3 serves  4 serves  5 serves or more | 70 (29.79)  119 (50.64)  30 (12.77)  13 (5.53)  3 (1.28) | 28 (26.17)  57 (53.27)  14 (13.08)  7 (6.54)  1 (0.93) | 42 (32.81)  62 (48.44)  16 (12.50)  6 (4.69)  2 (1.56) | 35 (31.53)  56 (50.45)  14 (12.61)  4 (3.60)  2 (1.80) | 35 (28.23)  63 (50.81)  16 (12.90)  9 (7.26)  1 (0.81) | 58 (31.02)  91 (48.66)  25 (13.37)  10 (5.35)  3 (1.60) | 11 (23.91)  27 (58.70)  5 (10.87)  3 (6.52)  0 (0.00) |
| **How often do you usually drink sugar sweetened drinks like soft drink, fruit drinks, added sugar fruit juice, cordials or sport drinks? ^2^** | | | | | | | |
| Every day or nearly every day  A few times a week  Once a week  Once a fortnight  Once a month  I don’t usually drink sugar sweetened drinks | 132 (55.46)  54 (22.69)  18 (7.56)  9 (3.78)  5 (2.10)  20 (8.40) | 62 (57.94)  22 (20.56)  8 (7.48)  5 (4.67)  2 (1.87)  8 (7.48) | 70 (53.44)  32 (24.43)  10 (7.63)  4 (3.05)  3 (2.29)  12 (9.16) | 75 (68.81)  23 (21.10)  4 (3.67)  3 (2.75)  1 (0.92)  3 (2.75) | 57 (44.19)  31 (24.03)  14 (10.85)  6 (4.65)  4 (3.10)  17 (13.18) | 108 (56.54)  45 (23.56)  15 (7.85)  6 (3.14)  2 (1.05)  15 (7.85) | 24 (53.33)  9 (20.00)  2 (4.44)  2 (4.44)  3 (6.67)  5 (11.11) |
| **How much do you usually drink?** | | | | | | | |
| 0.5 serve or less  0.5 to 1 serve  1 to 1.5 serves  1.5 to 2 serves  2 to 2.5 serves  2.5 to 3 serves  3 to 5 serves  5 serves or more | 1 (0.46)  27 (12.44)  38 (17.51)  30 (13.82)  22 (10.14)  16 (7.37)  39 (17.97)  44 (20.28) | 0 (0.00)  12 (12.12)  22 (22.22)  11 (11.11)  6 (6.06)  9 (9.09)  18 (18.18)  21 (21.21) | 1 (0.85)  15 (12.71)  16 (13.56)  19 (16.10)  16 (13.56)  7 (5.93)  21 (17.80)  23 (19.49) | 0 (0.00)  11 (10.48)  15 (14.29)  15 (14.29)  12 (11.43)  7 (6.67)  19 (18.10)  26 (24.76) | 1 (0.89)  16 (14.29)  23 (20.54)  15 (13.39)  10 (8.93)  9 (8.04)  20 (17.86)  18 (16.07) | 1 (0.57)  20 (11.43)  31 (17.71)  25 (14.29)  19 (10.86)  14 (8.00)  31 (17.71)  34 (19.43) | 0 (0.00)  6 (15.00)  6 (15.00)  5 (12.50)  3 (7.50)  2 (5.00)  8 (20.00)  10 (25.00) |
| **How often do you usually drink flavoured milk? ^2^** | | | | | | | |
| Every day or nearly every day  A few times a week  Once a week  Once a fortnight  Once a month  I don’t usually drink flavoured milk | 44 (18.26)  54 (22.41)  39 (16.18)  11 (4.56)  19 (7.88)  74 (30.71) | 25 (22.94)  24 (22.02)  16 (14.68)  4 (3.67)  5 (4.59)  35 (32.11) | 19 (14.39)  30 (22.73)  23 (17.42)  7 (5.30)  14 (10.61)  39 (29.55) | 25 (22.32)  25 (22.32)  22 (19.64)  8 (7.14)  9 (8.04)  23 (20.54) | 19 (14.73)  29 (22.48)  17 (13.18)  3 (2.33)  10 (7.75)  51 (39.53) | 39 (20.21)  44 (22.80)  28 (14.51)  8 (4.15)  17 (8.81)  57 (29.53) | 5 (10.87)  9 (19.57)  11 (23.91)  3 (6.52)  2 (4.35)  16 (34.78) |
| **How often do you usually have cheese or yoghurt?** | | | | | | | |
| Every day or nearly every day  A few times a week  Once a week  Once a fortnight  Once a month  I don’t usually eat cheese or yoghurt | 83 (34.44)  94 (39.00)  27 (11.20)  11 (4.56)  8 (3.32)  18 (7.47) | 36 (33.03)  40 (36.70)  12 (11.01)  5 (4.59)  4 (3.67)  12 (11.01) | 47 (35.61)  54 (40.91)  15 (11.36)  6 (4.55)  4 (3.03)  6 (4.55) | 35 (31.25)  45 (40.18)  15 (13.39)  4 (3.57)  4 (3.57)  9 (8.04) | 48 (37.21)  49 (37.98)  12 (9.30)  7 (5.43)  4 (3.10)  9 (6.98) | 70 (36.27)  74 (38.34)  22 (11.40)  9 (4.66)  6 (3.11)  12 (6.22) | 12 (26.09)  19 (41.30)  5 (10.87)  2 (4.35)  2 (4.35)  6 (13.04) |
| **If you have yoghurt, is it usually sweetened or unsweetened? ^2,3^** | | | | | | | |
| Sweetened/flavoured (vanilla-flavoured)  Unsweetened/plain | 105 (68.18)  49 (31.82) | 40 (63.49)  23 (36.51) | 65 (71.43)  26 (28.57) | 60 (76.92)  18 (23.08) | 45 (59.21)  31 (40.79) | 93 (72.09)  36 (27.91) | 12 (50.00)  12 (50.00) |
| **Do you drink plain milk nearly every day (includes milk added with cereal)? ^2^** | | | | | | | |
| Just in tea/coffee  No  Yes | 38 (15.77)  26 (10.79)  177 (73.44) | 12 (11.11)  12 (11.11)  84 (77.78) | 26 (19.55)  14 (10.53)  93 (69.92) | 13 (11.61)  18 (16.07)  81 (72.32) | 25 (19.38)  8 (6.20)  96 (74.42) | 33 (17.10)  24 (12.44)  136 (70.47) | 4 (8.70)  2 (4.35)  40 (86.96) |
| **How many serves of red meat (steak, beef, pork, kangaroo, lamb, sausages) do you usually eat per day?** | | | | | | | |
| Less than 1 serve  1 serve or more  I don’t eat red meat | 69 (28.51)  168 (69.42)  5 (2.07) | 30 (27.52)  78 (71.56)  1 (0.92) | 39 (29.32)  90 (67.67)  4 (3.01) | 26 (23.01)  84 (74.34)  3 (2.65) | 43 (33.33)  84 (65.12)  2 (1.55) | 58 (29.90)  132 (68.04)  4 (2.06) | 11 (23.91)  34 (73.91)  1 (2.17) |
| **When you eat red meat, do you usually cut the fat off?** | | | | | | | |
| No  Yes | 114 (48.72)  120 (51.28) | 59 (55.14)  48 (44.86) | 55 (43.31)  72 (56.69) | 59 (55.14)  48 (44.86) | 55 (43.31)  72 (56.69) | 89 (47.59)  98 (52.41) | 24 (53.33)  21 (46.67) |
| **How many serves of processed meat (devon, salami, sausage, ham, corned beef, pickled pork, rissole, smoked meat) do you usually eat per day? ^3^** | | | | | | | |
| Less than 1 serve  1 serve or more  I don’t eat processed meat | 71 (29.58)  162 (67.50)  7 (2.92) | 29 (26.85)  75 (69.44)  4 (3.70) | 42 (31.82)  87 (65.91)  3 (2.27) | 29 (25.89)  80 (71.43)  3 (2.68) | 42 (32.81)  82 (64.06)  4 (3.13) | 67 (34.90)  119 (61.98)  6 (3.13) | 4 (8.70)  41 (89.13)  1 (2.17) |
| **How many serves of white meat (chicken, turkey) do you usually eat per day?** | | | | | | | |
| Less than 1 serve  1 serve or more  I don’t eat white meat | 95 (39.42)  140 (58.09)  6 (2.49) | 48 (44.04)  58 (53.21)  3 (2.75) | 47 (35.61)  82 (62.12)  3 (2.27) | 38 (33.63)  71 (62.83)  4 (3.54) | 57 (44.53)  69 (53.91)  2 (1.56) | 82 (42.49)  106 (54.92)  5 (2.59) | 13 (28.26)  32 (69.57)  1 (2.17) |
| **When you eat chicken do you usually take the skin off? ^3^** | | | | | | | |
| No  Yes | 158 (68.10)  74 (31.90) | 77 (74.04)  27 (25.96) | 81 (63.28)  47 (36.72) | 73 (67.59)  35 (32.41) | 85 (68.55)  39 (31.45) | 117 (63.24)  68 (36.76) | 39 (86.67)  6 (13.33) |
| **Do you usually eat offal meat (such as kidney, liver, heart, brain, curly guts, moobal, tripe)? ^1,2^** | | | | | | | |
| No  Yes | 86 (35.83)  154 (64.17) | 28 (25.69)  81 (74.31) | 58 (44.27)  73 (55.73) | 53 (47.32)  59 (52.68) | 33 (25.78)  95 (74.22) | 70 (36.46)  122 (63.54) | 15 (32.61)  31 (67.39) |
| **How much butter, margarine or cream do you usually eat per day?** | | | | | | | |
| Less than 1 serve  1 serve or more  I don’t eat butter, margarine or cream | 70 (29.29)  160 (66.95)  9 (3.77) | 33 (30.28)  71 (65.14)  5 (4.59) | 37 (28.46)  89 (68.46)  4 (3.08) | 27 (24.32)  80 (72.07)  4 (3.60) | 43 (33.59)  80 (62.50)  5 (3.91) | 59 (30.89)  125 (65.45)  7 (3.66) | 10 (21.74)  34 (73.91)  2 (4.35) |
| **How much baked beans, three beans mix, cooked lentils, split peas or dried beans do you usually eat per week? ^2,3^** | | | | | | | |
| None  Less than 1 serve  1 to 2 serves  3 or more serves | 49 (20.42)  79 (32.92)  95 (39.58)  17 (7.08) | 23 (21.10)  28 (25.69)  48 (44.04)  10 (9.17) | 26 (19.85)  51 (38.93)  47 (35.88)  7 (5.34) | 30 (26.79)  40 (35.71)  34 (30.36)  8 (7.14) | 19 (14.84)  39 (30.47)  61 (47.66)  9 (7.03) | 44 (22.92)  64 (33.33)  68 (35.42)  16 (8.33) | 5 (10.87)  14 (30.43)  26 (56.52)  1 (2.17) |
| **How many eggs do you usually eat per week?** | | | | | | | |
| None  Less than 2 eggs  2 to 4 eggs  5 or more eggs | 8 (3.33)  37 (15.42)  100 (41.67)  95 (39.58) | 3 (2.75)  12 (11.01)  45 (41.28)  49 (44.95) | 5 (3.82)  25 (19.08)  55 (41.98)  46 (35.11) | 4 (3.57)  15 (13.39)  50 (44.64)  43 (38.39) | 4 (3.13)  22 (17.19)  50 (39.06)  52 (40.63) | 6 (3.13)  32 (16.67)  76 (39.58)  78 (40.63) | 2 (4.35)  5 (10.87)  23 (50.00)  16 (34.78) |
| **How much fish including wild caught fish, fresh and frozen seafood (including turtle, crayfish, yabbies, shrimp) do you usually eat per week (excluding fish fingers or fish patties)?** | | | | | | | |
| None  Less than 1 serve  1 to 2 serves  3 or more serves | 36 (15.06)  79 (33.05)  95 (39.75)  29 (12.13) | 16 (14.68)  34 (31.19)  41 (37.61)  18 (16.51) | 20 (15.38)  45 (34.62)  54 (41.54)  11 (8.46) | 18 (16.22)  39 (35.14)  45 (40.54)  9 (8.11) | 18 (14.06)  40 (31.25)  50 (39.06)  20 (15.63) | 33 (17.28)  64 (33.51)  71 (37.17)  23 (12.04) | 3 (6.52)  15 (32.61)  22 (47.83)  6 (13.04) |
| **How many times per week do you usually eat traditional native fruits, berries, nuts, or other wild plants?** | | | | | | | |
| None  Less than 1 time  1 to 2 times  3 or more times | 133 (55.65)  72 (30.13)  27 (11.30)  7 (2.93) | 55 (50.93)  33 (30.56)  15 (13.89)  5 (4.63) | 78 (59.54)  39 (29.77)  12 (9.16)  2 (1.53) | 66 (58.93)  32 (28.57)  11 (9.82)  3 (2.68) | 67 (52.76)  40 (31.50)  16 (12.60)  4 (3.15) | 114 (59.38)  52 (27.08)  19 (9.90)  7 (3.65) | 19 (42.22)  20 (44.44)  6 (13.33)  0 (0.00) |
| **How many times per week do you usually eat traditional meats like fish, kangaroo, emu, or goanna? ^1^** | | | | | | | |
| None  Less than 1 time  1 to 2 times  3 or more times | 112 (46.67)  74 (30.83)  43 (17.92)  11 (4.58) | 36 (33.03)  42 (38.53)  25 (22.94)  6 (5.50) | 76 (58.02)  32 (24.43)  18 (13.74)  5 (3.82) | 57 (50.89)  33 (29.46)  16 (14.29)  6 (5.36) | 55 (42.97)  41 (32.03)  27 (21.09)  5 (3.91) | 95 (49.48)  57 (29.69)  33 (17.19)  7 (3.65) | 16 (34.78)  17 (36.96)  9 (19.57)  4 (8.70) |
| **How many times per week do you usually eat unsalted nuts?** | | | | | | | |
| None  Less than 1 time  1 to 2 times  3 or more times | 155 (65.40)  45 (18.99)  25 (10.55)  12 (5.06) | 67 (61.47)  25 (22.94)  13 (11.93)  4 (3.67) | 88 (68.75)  20 (15.63)  12 (9.38)  8 (6.25) | 69 (62.73)  24 (21.82)  14 (12.73)  3 (2.73) | 86 (67.72)  21 (16.54)  11 (8.66)  9 (7.09) | 123 (64.74)  38 (20.00)  18 (9.47)  11 (5.79) | 32 (71.11)  7 (15.56)  5 (11.11)  1 (2.22) |
| **How many times per week do you usually eat pastries such as cookies, biscuits or cake?** | | | | | | | |
| None  Less than 1 time  1 to 2 times  3 or more times | 27 (11.25)  64 (26.67)  99 (41.25)  50 (20.83) | 15 (13.76)  29 (26.61)  36 (33.03)  29 (26.61) | 12 (9.16)  35 (26.72)  63 (48.09)  21 (16.03) | 14 (12.50)  31 (27.68)  48 (42.86)  19 (16.96) | 13 (10.16)  33 (25.78)  51 (39.84)  31 (24.22) | 22 (11.46)  53 (27.60)  76 (39.58)  41 (21.35) | 5 (10.87)  10 (21.74)  22 (47.83)  9 (19.57) |
| **How many times per week do you usually eat savoury snacks (e.g. chips, pretzels)? ^2,3^** | | | | | | | |
| None  Less than 1 time  1 to 2 times  3 or more times | 34 (14.23)  57 (23.85)  101 (42.26)  47 (19.67) | 13 (11.93)  23 (21.10)  49 (44.95)  24 (22.02) | 21 (16.15)  34 (26.15)  52 (40.00)  23 (17.69) | 6 (5.36)  23 (20.54)  51 (45.54)  32 (28.57) | 28 (22.05)  34 (26.77)  50 (39.37)  15 (11.81) | 23 (12.04)  50 (26.18)  76 (39.79)  42 (21.99) | 11 (23.91)  6 (13.04)  24 (52.17)  5 (10.87) |
| **How many times per week do you usually eat sweet snacks (e.g. lollies, chocolates or ice creams)? ^3^** | | | | | | | |
| None  Less than 1 time  1 to 2 times  3 or more times | 35 (14.52)  60 (24.90)  86 (35.68)  60 (24.90) | 16 (14.68)  24 (22.02)  43 (39.45)  26 (23.85) | 19 (14.39)  36 (27.27)  43 (32.58)  34 (25.76) | 10 (8.85)  28 (24.78)  45 (39.82)  30 (26.55) | 25 (19.53)  32 (25.00)  41 (32.03)  30 (23.44) | 21 (10.88)  57 (29.53)  65 (33.68)  50 (25.91) | 14 (30.43)  3 (6.52)  19 (41.30)  10 (21.74) |
| **How many times per week do you usually eat store-bought ready meals (e.g. supermarket pizza, instant noodles)? ^1,2,3^** | | | | | | | |
| None  Less than 1 time  1 to 2 times  3 or more times | 64 (26.67)  60 (25.00)  87 (36.25)  29 (12.08) | 21 (19.27)  27 (24.77)  49 (44.95)  12 (11.01) | 43 (32.82)  33 (25.19)  38 (29.01)  17 (12.98) | 16 (14.16)  27 (23.89)  47 (41.59)  23 (20.35) | 48 (37.80)  33 (25.98)  40 (31.50)  6 (4.72) | 56 (29.17)  49 (25.52)  61 (31.77)  26 (13.54) | 8 (17.39)  10 (21.74)  25 (54.35)  3 (6.52) |
| **How many times per week do you usually eat meals or snacks from take-away such as pizza, burgers, fries, pies, fish and chips? ^2^** | | | | | | | |
| None  Less than 1 time  1 to 2 times  3 or more times | 37 (15.42)  88 (36.67)  94 (39.17)  21 (8.75) | 16 (14.68)  34 (31.19)  47 (43.12)  12 (11.01) | 21 (16.03)  54 (41.22)  47 (35.88)  9 (6.87) | 9 (8.04)  34 (30.36)  55 (49.11)  14 (12.50) | 28 (21.88)  54 (42.19)  39 (30.47)  7 (5.47) | 27 (14.06)  66 (34.38)  79 (41.15)  20 (10.42) | 10 (21.74)  21 (45.65)  14 (30.43)  1 (2.17) |
| **How many times per week do you usually use salty seasonings/sauces for vegetables, pasta, rice or other dishes?** | | | | | | | |
| None  Less than 1 time  1 to 2 times  3 or more times | 21 (8.82)  44 (18.49)  112 (47.06)  61 (25.63) | 10 (9.35)  18 (16.82)  47 (43.93)  32 (29.91) | 11 (8.40)  26 (19.85)  65 (49.62)  29 (22.14) | 5 (4.50)  24 (21.62)  50 (45.05)  32 (28.83) | 16 (12.60)  20 (15.75)  62 (48.82)  29 (22.83) | 16 (8.42)  36 (18.95)  93 (48.95)  45 (23.68) | 5 (10.87)  7 (15.22)  18 (39.13)  16 (34.78) |
| **How often do you eat bread and cereals (e.g. breakfast cereals, bread, scones, jonny cakes, damper, rice, pasta, other grains)?** | | | | | | | |
| Every day or nearly every day  A few times a week  Once a week  Once a fortnight  Once a month  I don’t eat bread and cereals | 186 (77.50)  39 (16.25)  10 (4.17)  5 (2.08)  0 (0.00)  0 (0.00) | 90 (82.57)  14 (12.84)  5 (4.59)  0 (0.00)  0 (0.00)  0 (0.00) | 96 (73.28)  25 (19.08)  5 (3.82)  5 (3.82)  0 (0.00)  0 (0.00) | 89 (79.46)  19 (16.96)  2 (1.79)  2 (1.79)  0 (0.00)  0 (0.00) | 97 (75.78)  20 (15.63)  8 (6.25)  3 (2.34)  0 (0.00)  0 (0.00) | 145 (75.52)  35 (18.23)  9 (4.69)  3 (1.56)  0 (0.00)  0 (0.00) | 40 (86.96)  3 (6.52)  1 (2.17)  2 (4.35)  0 (0.00)  0 (0.00) |
| **When you eat bread and cereals, how much do you usually eat per day? ^1^** | | | | | | | |
| 1 serve or less  2 to 3 serves  4 to 5 serves  6 to 7 serves  8 serves or more | 36 (15.06)  111 (46.44)  52 (21.76)  25 (10.46)  15 (6.28) | 11 (10.09)  42 (38.53)  33 (30.28)  17 (15.60)  6 (5.50) | 25 (19.23)  69 (53.08)  19 (14.62)  8 (6.15)  9 (6.92) | 16 (14.29)  48 (42.86)  29 (25.89)  12 (10.71)  7 (6.25) | 20 (15.75)  63 (49.61)  23 (18.11)  13 (10.24)  8 (6.30) | 31 (16.23)  88 (46.07)  41 (21.47)  17 (8.90)  14 (7.33) | 4 (8.70)  23 (50.00)  11 (23.91)  7 (15.22)  1 (2.17) |
| **What type of bread do you usually eat?** | | | | | | | |
| White ^**^ | 177 (73.14) | 78 (71.56) | 99 (74.44) | 93 (82.30) | 84 (65.12) | 142 (73.20) | 35 (76.09) |
| Multigrain/rye/wholemeal | 118 (48.76) | 56 (51.38) | 62 (46.62) | 50 (44.25) | 68 (52.71) | 98 (50.52) | 18 (39.13) |

^a^ Analysis by location excludes two respondents with unknown location

^b^ Other areas include Gingie Reserve, Namoi Village, and out of town Walgett area

^*^ Significant difference by sex at p<0.05.

^**^ Significant difference by age group at p<0.05.

^***^ Significant difference by location at p<0.05.
